# Collaborative management of the Grand Ethiopian Renaissance Dam increases economic benefits and resilience

**DOI:** 10.1038/s41467-021-25877-w

**Published:** 2021-09-23

**Authors:** Mohammed Basheer, Victor Nechifor, Alvaro Calzadilla, Khalid Siddig, Mikiyas Etichia, Dale Whittington, David Hulme, Julien J. Harou

**Affiliations:** 1 Department of Mechanical, Aerospace and Civil Engineering, The University of Manchester, Manchester, UK; 2 Institute for Sustainable Resources, University College London, London, UK; 3 International Agricultural Trade and Development, Humboldt-Universität zu Berlin, Berlin, Germany; 4 Department of Agricultural Economics, University of Khartoum, Khartoum, Sudan; 5 Global Development Institute, The University of Manchester, Manchester, UK; 6 Departments of Environmental Sciences and Engineering and City and Regional Planning, University of North Carolina, Chapel Hill, NC USA; 7 Department of Civil, Environmental and Geomatic Engineering, University College London, London, UK

**Keywords:** Hydrology, Energy infrastructure, Economics, Water resources

## Abstract

The landscape of water infrastructure in the Nile Basin is changing with the construction of the Grand Ethiopian Renaissance Dam. Although this dam could improve electricity supply in Ethiopia and its neighbors, there is a lack of consensus between Ethiopia, Sudan, and Egypt on the dam operation. We introduce a new modeling framework that simulates the Nile River System and Egypt’s macroeconomy, with dynamic feedbacks between the river system and the macroeconomy. Because the two systems “coevolve” throughout multi-year simulations, we term this a “coevolutionary” modeling framework. The framework is used to demonstrate that a coordinated operating strategy could allow the Grand Ethiopian Renaissance Dam to help meet water demands in Egypt during periods of water scarcity and increase hydropower generation and storage in Ethiopia during high flows. Here we show the hydrological and macroeconomic performance of this coordinated strategy compared to a strategy that resembles a recent draft proposal for the operation of the dam discussed in Washington DC.

## Abstract (web summary)

Integrating river system and economy-wide models in a dynamic, iterative, bidirectional fashion allows assessing some economic impacts of interventions in river systems. Here the authors use this framework to compare water resources management strategies for the Nile in a quest for efficient use of the river’s limited and stressed water resources.

## Introduction

Freshwater and electricity are essential inputs to many production activities that drive the economic development and well-being of societies. The scarcity and variability of freshwater resources have been shown to affect the economic growth of nations^1,2^. Empirical evidence of uni- and bidirectional relationships between energy consumption and economic development have been documented in countries around the world^3–6^. Water and energy systems are interlinked with each other and with several sectors, including agriculture and industry^7^. Globally, hydropower contributes around 16% of electricity generation and approximately 70% of renewable electricity generation^8^, and rivers are frequently used to cool power plants^9,10^. Energy is used for water treatment, pumping, and desalinization. Therefore, efficient use of limited water resources to achieve sustainable economic development requires assessing water and economy interventions in an integrated way.

**Fig. 1:**
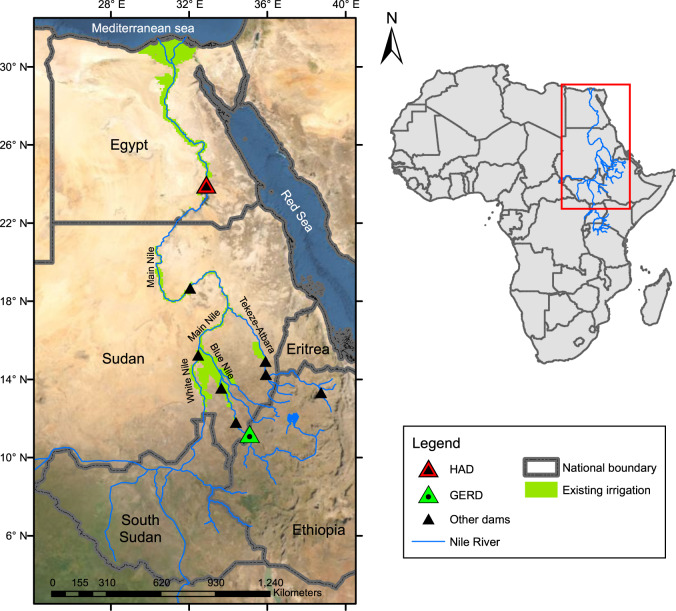
Major dams and existing large-scale irrigation in the Eastern Nile River Basin. The true-color image shown in the figure’s background is based on the satellite imagery of the world managed by the Environmental Systems Research Institute (ESRI). The existing irrigation layer is based on the Global Map of Irrigation Areas (GMIA) developed by the Food and Agriculture Organization (FAO) of the United Nations. The national boundary layer is based on the Database of Global Administrative Areas (GADM).

The Nile is one of the longest rivers in the world and has a basin that extends over 11 African countries, each with a different contribution to and economic dependence on the river^11,12^. The Nile comprises three main tributaries: the White Nile, the Blue Nile, and the Tekeze-Atbara (see Fig. 1). The Blue Nile originates in Ethiopia and contributes around 57% of the Nile streamflow as measured near the Sudanese–Egyptian border^13^. High inter- and intra-annual variabilities characterize the Blue Nile streamflow, with around 80% of the flow occurring from July to October^14^. Nearly all the Nile streamflow, measured near the Sudanese–Egyptian border, is currently consumed by the two most downstream riparian countries, i.e., Egypt and Sudan. Egypt’s water, energy, food, and economic system is linked to the Nile streamflow, which provides around 90% of the country’s freshwater consumption^15^ and 7% of its electricity supply through hydropower^8^. On average, irrigated agriculture accounts for approximately 82% of Egypt’s annual Nile water withdrawal, while municipal and industrial water users account for 18%^16^.

In 1999, the Nile Basin countries established the Nile Basin Initiative (NBI) as a forum for coordination and collaboration on managing the river^17^. The NBI refers to two regions of the Nile Basin: the Eastern Nile Basin (Fig. 1) and the Nile Equatorial Lakes Region. The NBI worked with the member states to craft the Nile River Basin Cooperative Framework Agreement (CFA)^18^. In 2010, Egypt and Sudan froze their memberships in the NBI due to disagreements over the text of the CFA, but Sudan returned to full membership 2 years later^19^.

The United States Bureau of Reclamation conducted a study between 1958 and 1963 (published in 1964) that identified potential dams and irrigation projects on the Blue Nile in Ethiopia^20^. Dams on the Blue Nile in Ethiopia could increase hydropower generation markedly^21^. However, they could increase the complexity of managing multiyear droughts^20,22^. Under the auspices of the NBI, Ethiopia, Sudan, and Egypt launched the Joint Multipurpose Program in 2005 to facilitate coordinated development of investment projects in the Eastern Nile Basin. The three countries invited the World Bank to constitute an independent expert team to conduct a scoping study to inform the selection of a first set of multipurpose projects that would benefit all three countries^22^. The scoping study team concluded that the best investment opportunities for a joint multipurpose project were in the Blue Nile Basin in Ethiopia. Egyptian policymakers and technical experts contested this conclusion and argued that there were promising options for a joint multipurpose project in the Baro–Akobo–Sobat Basin^23^. This disagreement on the scoping study’s main conclusion led to a loss of political momentum and eventually failure to achieve the JMP goals^23^. In 2011, Ethiopia unilaterally started the construction of the Grand Ethiopian Renaissance Dam (GERD). On 12 July 2020, construction was around 75% complete, and Ethiopia began the initial filling of the dam’s reservoir.

When completed, the GERD will be the largest hydropower facility in Africa, with a power capacity of 5150 MW and reservoir storage of 74 billion cubic meters (bcm). The dam will double Ethiopia’s electricity generation and potentially stimulate the country’s economic growth through increases in the output of electricity-dependent sectors and other sectors via forward and backward economic linkages. However, the initial filling and long-term operation of the GERD reservoir are expected to significantly alter the pattern of flow of the Blue Nile downstream of the dam, imposing a range of opportunities and risks to Sudan and Egypt^24–26^. Sudan is expected to benefit from the GERD in terms of improved irrigation water supply reliability, hydropower generation, and riverine flood control provided there is essential daily coordination and data sharing with Ethiopia^24,27^, but adverse environmental impacts and a loss of recession agriculture are also anticipated^28^.

Despite several years of negotiations (since 2011) between the Ethiopian, Sudanese, and Egyptian governments on the GERD’s initial filling and long-term operation, no agreement has yet been reached. In 2012, the three countries formed an International Panel of Experts (IPoE) to review the design and impact reports of the dam^29^. The IPoE recommended conducting further technical studies on the GERD design and impacts. An international, non-partisan Eastern Nile working group met at the Massachusetts Institute of Technology in 2014 to discuss the impacts of the GERD on regional development^30^. The group pointed out four issues: the need for coordinated operation of the GERD and the High Aswan Dam (HAD), technical concerns on the design of the GERD, the need for an electricity sale agreement, and potential negative impacts on irrigated agriculture in Egypt and recession agriculture in Sudan. In March 2015, the heads of the Ethiopian, Sudanese, and Egyptian governments signed a Declaration of Principles (DoP) on the GERD^31^. Most of the principles in the DoP are derived from the United Nations Convention on the Law of the Non-navigational Uses of International Watercourses^32^, including the principles of equitable and reasonable utilization and not to cause significant harm. The DoP stated the need to conduct the studies recommended by the IPoE and utilize the outcomes of these studies to agree on rules and guidelines for the initial filling and long-term operation of the GERD. In 2015, the three countries agreed to contract a consortium of international consultancy firms to conduct the studies recommended by the IPoE. However, this effort failed due to disagreements among the riparians on the terms of reference of the studies and how baseline water allocations should be handled in the construction of scenarios to be examined in the consortium’s analyses^33^.

In 2018, Ethiopia, Sudan, and Egypt formed a National Independent Scientific Research Group (NISRG) of researchers from the three countries. The NISRG process did not lead to a final agreement among the riparians, but its technical outcomes constituted the basis for subsequent negotiations on the initial filling and long-term operation of the dam^34^. From November 2019 to February 2020, several rounds of negotiations occurred, including meetings in Washington DC with the United States Government and the World Bank as observers^35^; yet, no agreement was signed. From June 2020, the African Union initiated and hosted further negotiations between Ethiopia, Sudan, and Egypt with the United States Government, the World Bank, and the European Union as observers. Still, no agreement had been reached at the time this article was being finalized (September 2021). The main points of contention remaining among the riparians are (1) the length of the agreement, i.e., whether it is an interim or permanent agreement; (2) the relationship between the GERD agreement and future water development projects in Ethiopia, (3) the linkage between the GERD agreement and a permanent water allocation agreement among the riparians in the Nile Basin, and (4) the mechanisms to resolve future conflicts should they arise^34^.

Previous studies have investigated the impacts of GERD filling and long-term operation on Ethiopia, Sudan, and Egypt^24–27,36–40^. However, these studies used simple representations of the linkages between the river system and the Eastern Nile economies. In reality, economic growth affects water and electricity demands, and the abundance or scarcity of water and electricity have a feedback effect on economic growth^41^.

We have two objectives in this paper. The first is to present a new coevolutionary hydro-economic modeling framework that captures the dynamic interactions between a river’s hydrology and infrastructure and the macroeconomy of one of the river’s riparians (Egypt). The second is to use this multi-sector dynamic modeling framework to examine a coordinated operating strategy (termed “coordinated operation”) for filling and operating the GERD on the Nile River. With the coordinated operation, the GERD helps, under specific conditions, meet water consumption targets in Egypt and Sudan, and Ethiopia takes advantage of extra water during periods of high flows to increase GERD storage and maximize hydropower production. Coordinated operation is best conceptualized as an operating policy of “neighbors looking out for each other,” especially during multiyear hydrological droughts. We compare the coordinated operation strategy to a strategy that resembles the proposal for operating the GERD discussed as part of the negotiations between Egypt, Sudan, and Ethiopia in late 2019 and early 2020 in Washington DC (herein, the examined strategy, including some assumptions, is termed the “Washington draft proposal”). Details on the assumptions and differences between coordinated operation and the GERD operating policy in the Washington draft proposal are provided in the next section and the “Methods” section. To assess the dynamic interactions between the Nile and the sectors of Egypt’s economy, we use a calibrated river system model of the Eastern Nile Basin coupled with a Computable General Equilibrium (CGE) model of Egypt’s economy. The water and economy models are developed and connected using open-source modeling frameworks^42–44^, as described in the “Methods” section. Results show that in most of the examined hydrological scenarios, coordinated filling and operation of the GERD increases the total electricity generation from both the GERD and the entire Nile system, sustains Sudan’s Nile water use, decreases Egypt’s irrigation water deficits, and increases Egypt’s total gross domestic product (GDP) and other macroeconomic metrics compared to the Washington draft proposal.

## Results

### The Washington draft proposal versus coordinated operation

We compare the impacts of two GERD filling and long-term operation approaches: (a) Washington draft proposal and (b) coordinated operation. Table 1 summarizes the two examined operating strategies and their key assumptions. Further details on how the two operating strategies are implemented in the modeling framework are provided in the “Methods” section. Both operating strategies assume that Ethiopia targets withdrawal of 2.5 bcm annually upstream of the GERD and that Sudan targets withdrawal of 17.7 bcm/year. The assumed total Ethiopian water withdrawal target is the sum of the withdrawal targets of the Finchaa and Beles irrigation schemes, which are on the Blue Nile, whereas the total Sudanese water withdrawal target is the sum of the withdrawal targets of existing irrigation and municipal water users in Sudan on the Blue Nile, the White Nile, the Tekeze-Atbara, and the Main Nile. Egypt attempts to withdraw 3.8 bcm upstream of the HAD and release 51.7 bcm from the HAD (a total of 55.5 bcm), its water allocation under the 1959 Nile Waters Agreement^45^. Deficits in Egypt are measured from this 55.5 bcm target. It is worth noting that Egypt and Sudan have different views on how evaporation losses should be considered in their 1959 bi-lateral water allocation agreement^45^. Egypt believes that reservoir evaporation from dams constructed after the 1959 agreement (i.e., Merowe, Roseires heightening, and Upper Atbara and Setit) is part of water allocations, while Sudan argues the opposite^25^. In this study, we assume the target withdrawals by Ethiopia and Sudan and the target releases from the HAD to illustrate the behavior of the hydrological and economic systems; they do not reflect an endorsement of the status quo water allocation in the Nile Basin.

**Table 1:** Main assumptions and attributes of the Washington draft proposal and coordinated operation.

Attribute	Washington draft proposal	Coordinated operation
Water withdrawal targets (bcm)	Ethiopia = 2.5 Sudan = 17.7	Ethiopia = 2.5 Sudan = 17.7
Water withdrawal target from the HAD reservoir plus water release target from the HAD (bcm)	Egypt = 55.5	Egypt = 55.5
GERD water release rules	• Complying with drought mitigation mechanisms (see the “Methods” section for details) • Generating 1170 GWh/month during long-term operation when reservoir storage is at or above 49.3 bcm • Generating 585 GWh/month during long-term operation when reservoir storage is below 49.3 bcm	• When physically possible, monthly releases are greater than or equal to Sudan’s monthly water withdrawal targets on the Blue and Main Nile plus Egypt’s monthly water release targets from HAD if HAD storage is below 50 bcm (156 m a.s.l.) • Generating 1170 GWh/month during long-term operation when reservoir storage is at or above 49.3 bcm • Generating 585 GWh/month during long-term operation when reservoir storage is below 49.3 bcm
Roseires, Sennar, and Merowe dams water release rules	• During GERD filling, water releases are according to historical rules • During GERD long-term operation, reservoirs are kept as high as possible to maximize energy generation	• During GERD filling, water releases are according to historical rules. • During GERD long-term operation, reservoirs are kept as high as possible to maximize energy generation • Making way for water releases from the GERD that are intended to benefit Egypt
HAD water release rules	• When Egypt’s drought management plan is not invoked, water is released to meet downstream demands • Water releases for irrigation are reduced according to a drought management plan if the reservoir storage is ≤60 bcm (approximately 160 m a.s.l.)	• When Egypt’s drought management plan is not invoked, water is released to meet downstream demands • Water releases for irrigation are reduced according to a drought management plan if the reservoir storage is ≤60 bcm (approximately 160 m a.s.l.)

In this study, the examined Washington draft proposal resembles the proposal annexed in the letter of the permanent representative of Egypt to the United Nations to the President of the United Nations Security Council dated 19 June 2020^46^. Ethiopia has not accepted this draft proposal. The Washington draft proposal suggests limiting the period during which Ethiopia can retain Nile flows to fill the GERD Reservoir to the peak of the annual flood season in July and August^35^. The Washington draft proposal enables Ethiopia to ramp up GERD storage so that all turbines can become operational within the first 2 years of initial filling to guarantee that Ethiopia can quickly begin electricity generation. The Washington draft proposal mitigates the consequences of droughts, prolonged droughts, and prolonged periods of dry years using three operating constraints on the GERD^46^: (a) a minimum annual release depending on the inflow, (b) a 4-year minimum average annual release, and (c) a 5-year minimum average annual release. Given these three constraints on GERD operation, Ethiopia would still have some operational flexibility. Thus, we assume that once reservoir storage reaches the long-term operation stage (49.3 bcm), Ethiopia would operate the GERD to maximize the 90% power reliability and sustain a minimum environmental flow of 43 Mm^3^/day (million cubic meters/day) subject to these three drought mitigation mechanisms. Further details on the drought mitigation mechanisms of the Washington draft proposal and their implementation and assumptions in the model are provided in the “Methods” section.

**Fig. 2:**
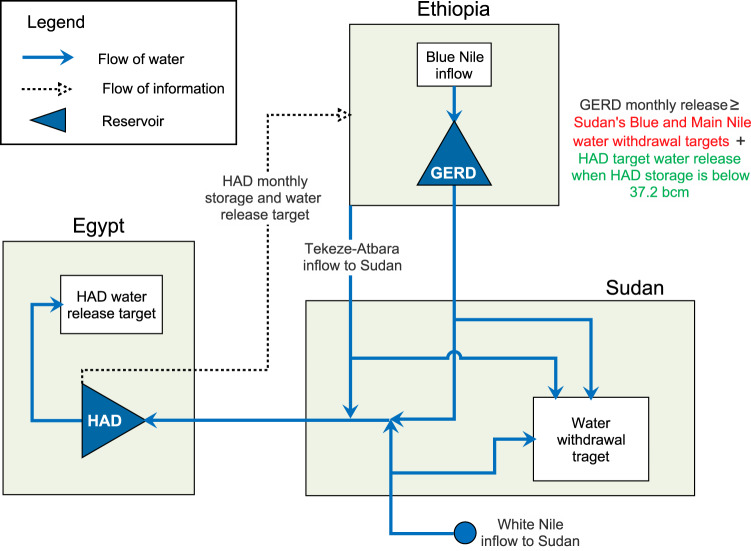
Illustration of the coordinated strategy for operating the Grand Ethiopian Renaissance Dam. HAD High Aswan Dam, GERD Grand Ethiopian Renaissance Dam. A detailed schematic of the river system model is provided in Supplementary Figure 7.

Figure 2 shows a flowchart of the examined coordinated operation strategy. A description and a schematic of the Eastern Nile River system model are provided in the “Methods” section and the Supplementary information. The rationale behind the coordinated operation strategy we examine in this study aims for some measure of hydro-solidarity, “neighbors looking out for each other,” where political boundaries are relaxed but with some national goals remaining. The coordinated operation strategy is not designed to be a prescribed solution for Nile water issues; rather, we use it to demonstrate the direction of change in economic performance and resilience that transboundary collaboration holds. We configured the coordinated operation strategy manually through trial and error, inspired by operating policies of previous studies^24,37,47^. In the coordinated operation approach, each of the three countries has a role to play. Ethiopia has more flexibility in GERD management when there is sufficient water in the HAD reservoir (HADR) and when the flows of the other two Eastern Nile tributaries (i.e., the White Nile and Tekeze-Atabra; Fig. 1) are high. In addition, Egypt shares information with Ethiopia on HAD storage and target water release. Coordinated operation enables Ethiopia to avoid the constraints on minimum releases from the GERD that are part of the Washington draft proposal. Instead, water releases from the GERD always ensure the satisfaction of water consumption targets on the Blue Nile and Main Nile in Sudan, and when physically possible and HAD storage is low (below 50 bcm), the satisfaction of water consumption targets in Egypt, with Ethiopia able to seek additional benefits under favorable streamflow conditions. A HADR storage of 50 bcm is equivalent to a reservoir water level of 156 m a.s.l., which is 9 m above the turbine shutdown level of the dam^24^. With thecoordinated operation, the operations of the Roseires, Sennar, and Merowe dams located in Sudan between the GERD and Egypt have been adapted to pass water releases from the GERD intended to reach Egypt. Ethiopia is assumed to operate the GERD to maximize the 90% power reliability and sustain a minimum environmental flow of 43 Mm^3^/day, subject to constraints that water consumption targets in Sudan and Egypt are satisfied under specific conditions.

These two filling and long-term operation approaches are analyzed using 102 different 30-year river flow sequences (traces) developed using the index-sequential method^48^. This method generates river flow traces from the historical flow record, taking every year in the record as a possible starting point. We used the 1901–2002 Nile flow data to generate the river flow traces (see Supplementary Fig. 1).

Sudan receives irrigation, flood control, and hydropower benefits from the GERD, assuming daily coordination and active data sharing between the GERD and Roseires Dam. The modeling results show that these benefits are essentially the same with the Washington draft proposal and the coordinated operation. This is due to Sudan’s geographic advantage of being located upstream of Egypt and the relatively small storage dams and hydropower capacity in the country. Furthermore, we assume that the adverse environmental impacts and the loss of recession agriculture in Sudan are similar in the two examined GERD operation scenarios. Therefore, we only present the results of the impacts of the coordinated operation for Ethiopia and Egypt.

### Coordination can improve water utilization

**Fig. 3:**
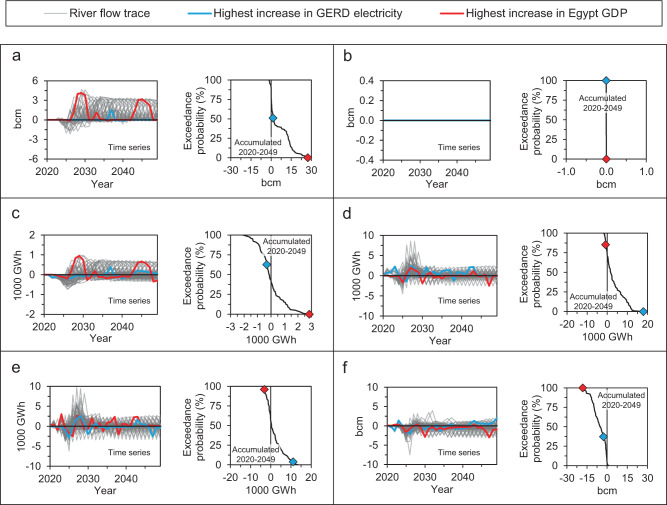
Difference in simulated water and energy metrics between the coordinated operation and the Washington draft proposal. **a** Irrigation water withdrawal in Egypt. **b** Municipal water withdrawal in Egypt. **c** Hydropower generation in Egypt. **d** Electricity generation from the Grand Ethiopian Renaissance Dam. **e** System-wide electricity generation from hydropower. **f** Total evaporation from human-made reservoirs, Toshka spills, and river channel seepage. Positive values indicate higher simulated results with coordinated operation. The cyan- and red-colored points in the probability plots correspond to the cyan- and red-colored river flow traces, respectively. GDP gross domestic product, GERD Grand Ethiopian Renaissance Dam.

Figure 3 illustrates the change in Nile water withdrawal in Egypt, hydropower generation of the GERD and Egypt, and the total reservoir evaporation, Toshka spills, and river channel seepage as a result of coordinated operation compared to the Washington draft proposal. Table 2 reports statistics for some of the metrics shown in Fig. 3. Results show that in 77% of the traces simulated,  the coordinated operation would decrease Egypt’s total water deficits compared to the Washington draft proposal. Most of the significant decreases in irrigation water deficits occur after 2025 because the HADR is currently full^49^ and can satisfy any near-term Egyptian water supply deficits that may occur in a specific simulation. Supplementary Fig. 2 shows that the decreases in Egypt’s irrigation deficits occur during multiyear periods of water scarcity. Supplementary Fig. 2 also shows a drawdown of GERD storage with the coordinated operation to help alleviate irrigation water deficits in Egypt when HAD storage falls below 50 bcm. This decline in GERD storage resulted in a small reduction in the dam’s total energy generation.

**Table 2:** Difference in selected water, energy, and economy metrics between the coordinated operation and the Washington draft proposal.

Metrics accumulated over 30 years	Maximum	90th percentile	Median	10th percentile	Minimum
Change in irrigation water withdrawal in Egypt (bcm)	27.3	16.2	1.4	0.0	−1.7
Change in Egypt hydropower generation (1000 GWh)	2.7	1.2	−0.1	−1.0	−2.1
Change in GERD electricity generation (1000 GWh)	17.9	10.5	1.6	−0.8	−1.9
Change in water losses (bcm)	1.1	−0.2	−5.1	−12.1	−18.1
Change in Egyptian GDP present value (US$ billion)	4.1	2.5	0.24	0.0	−0.7
Change in present value of GERD electricity (US$ billion)	0.6	0.4	0.06	0.0	−0.05

Positive values indicate higher simulated results with the coordinated operation.

Over 2020–2049, 58% of modeled traces show a decrease in the total Egyptian hydropower generation in the coordinated operating strategy compared to the Washington draft proposal. Most of the significant annual declines in Egypt’s hydropower happen from 2020 to 2030 due to the faster decrease in HADR storage with the coordinated operation that results from rapid GERD initial filling, which is enabled by the HADR having enough initial storage at the start of the simulation to meet Egypt’s target releases. In some simulated traces, electricity generation from the HAD also declines beyond 2030 with the coordinated operation compared to the Washington draft proposal. This occurs because, in some simulated traces, the coordinated operation shifts water storage from the HADR to GERD Reservoir, resulting in lower water levels at the HAD and higher water levels at the GERD.

Figures 3d, e show the effect of the coordinated operation on GERD and system-wide hydropower generation, respectively, compared to the Washington draft proposal. The time-series plots show some declines in GERD electricity within the first 5 years, followed by increases. The declines occur because of lower downstream water releases in the first 5 years to speed up reservoir filling when the storage in the HADR is sufficient to supplement river flows and thus avoid irrigation deficits in Egypt. The long-term increase in GERD electricity generation (e.g., the cyan-colored line in Fig. 3d) results from the faster initial filling and the opportunistic long-term management of dam releases in coordination with Egypt and Sudan. Supplementary Fig. 3 shows how coordinated operation can result in higher reservoir storage at GERD without adversely affecting Egypt’s water consumers. Seventy-one percent of the simulated traces show an increase in the GERD’s cumulative electricity generation over 2020–2049 compared to the Washington draft proposal. The change in the GERD’s total electricity generation over the 2020–2049 simulation period ranges from −1900 to 17,900 GWh, with a median of around 1600 GWh. The changes in the GERD’s annual electricity range from −35 to 84% compared to the operating policy of the Washington draft proposal, with a median annual change of 0%, increases in 40% of the years, and decreases in 10% of the years. Figure 3f shows the impact of the coordinated operation on total water losses from reservoir evaporation, spills to the Toshka depression in Egypt, and channel seepage compared to the Washington draft proposal. The coordinated operation changed cumulative water losses over the 30-year simulation by −18.1 to 1.1 bcm depending on the hydrological trace, with a median of around −5.1 bcm.

### Coordination can enhance economic resilience

**Fig. 4:**
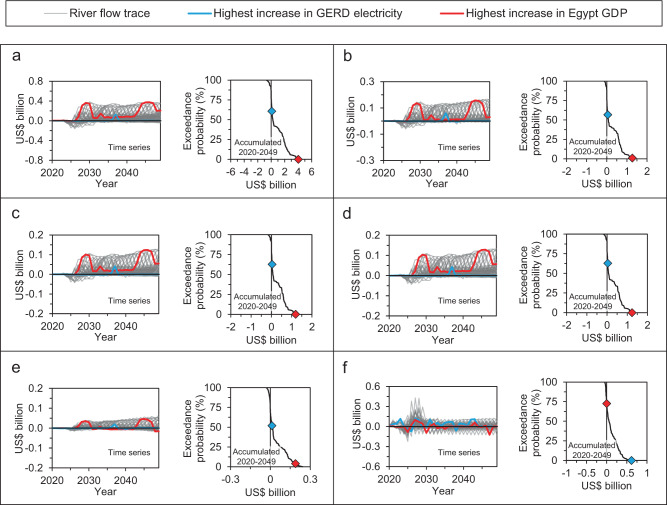
Difference in simulated economic metrics of Egypt and Ethiopia between the coordinated operation and the Washington draft proposal. **a** Egypt’s GDP at market prices. **b** Egypt’s investment. **c** Egypt’s export. **d** Egypt’s imports. **e** Egypt’s government savings. **f** The financial value of GERD electricity. Positive values indicate higher simulated results with the coordinated operation. The cyan- and red-colored points in the probability plots correspond to the cyan- and red-colored river flow traces, respectively. The results are discounted at a 3% rate. The results in panel (**f**) are based on Ethiopia’s current electricity export price to Sudan (US$ 0.05 per kWh). GDP gross domestic product, GERD Grand Ethiopian Renaissance Dam.

Nile flows to Egypt play a vital role in the country’s economy. The agriculture sector accounts for around 23% of the country’s employment^50^. Changes to irrigation water supply affect the output of agriculture and the livelihoods and employment of millions of Egyptians. Moreover, changes to irrigation water availability affect other economic activities that use agricultural products as intermediate inputs due to forward and backward economic linkages. Although hydropower contributes only around 7% to the Egyptian electricity mix, reduction in hydropower generation increases the use of other electricity generation technologies with higher variable costs. Figure 4a–e depicts the change in some macroeconomic metrics of the Egyptian economy as a result of  the coordinated operation compared to the Washington draft proposal. Table 2 reports key statistics of some of these metrics from the model simulations. Overall, the Egyptian GDP, investment, exports, imports, and government savings would increase as a result of the coordinated operation. The first 5 years of all model simulations show an insignificant change in macroeconomic performance due to the coordinated operation because the HADR starts the simulation full and can supplement reduced inflows to the HADR due to GERD filling and thus Egypt’s water needs can continue to be met. The slight decline in macroeconomic performance during the filling period is due to a reduction in Egypt’s hydropower generation as a result of a speedup of GERD filling under coordinated operation compared to the Washington draft proposal (Fig. 3c). The positive changes in Egyptian macroeconomic performance beyond 2030 are due to improved irrigation water supply.

As the economy-wide modeling results show, the direct and indirect impacts of reduced irrigation deficits increase the production of all economic activities and increase investment (Fig. 4b). Investment grows due to an increase in the savings of households, enterprises, and the Government of Egypt. Imports and exports also increase with coordinated filling and long-term operation of the GERD (Figs. 4c, d). Results for the coordinated operation show an increase in government savings compared to the Washington draft proposal (Fig. 4e). Government income increases as a result of the indirect impacts of improvements in irrigation water supply. Fewer and smaller irrigation water supply deficits lead to an increase in the production of many industries, which increases tax revenues. Moreover, the increase in industrial production raises household income and demand for commodities, imports, and import duties. Government savings increase with coordinated operation as a result of the increase in government income. With the coordinated operation, government spending increases; the increase in government spending is lower than the increase in government income resulting in a net positive increase in government savings. Results show that the change in the present value of Egypt’s GDP over the 2020–2049 simulated traces ranges between US$ −0.7 and US$ 4.1 billion with a median of around US$ 0.24 billion (at a 3% discount rate) and an increase in about 76% of the examined hydrologic scenarios. Egypt’s investment, exports, imports, and government savings follow a similar pattern to that of GDP with median present value changes of around US$ 80, 70, 70, and 20 million, respectively, if the coordinated operation is adopted. Most of the improvements in Egypt’s macroeconomic performance occur during multiyear periods of water scarcity (5–15 years of the 30-year simulation period) when HAD storage is below 50 bcm. The low median change in economic performance indicates that in a large proportion of the simulated traces, the HADR does not drop to a level that triggers GERD’s help.

Figure 4f shows the present value of the change in the GERD’s electricity generation as a result of coordinated operation compared to the Washington draft proposal. These results assume that the change in electricity is valued at Ethiopia’s current electricity export price to Sudan (US$ 0.05 per kilowatt-hour (kWh)). It would be possible to estimate both the direct and indirect effects of the GERD’s hydropower generation on the Ethiopian economy using a CGE model similar to that used for the Egyptian economy. However, a lack of information about how much of the dam’s electricity will be consumed domestically, how much will be exported, and the price at which electricity exported is sold made this approach impractical in our analysis. Figure 4f shows the change in the present value of the GERD’s electricity generation due to coordinated operation over the period 2020–2049, assuming a value per kilowatt-hour of US$ 0.05 and a discount rate of 3%. The coordinated operation results in a median change in the present value of the GERD’s electricity generation of US$ 0.06 billion (range of minus US$ 0.05 billion to US$ 0.60 billion depending on the hydrological scenario). Coordinated operation results in an increase in the present value of the GERD’s electricity generation in 71% of the examined hydrological scenarios. The coordinated operation thus yields improved economic outcomes to both Egypt and Ethiopia compared to the Washington draft proposal.

## Discussion

River flow is a complex, stochastic process that cannot be accurately predicted^51^. Our analysis shows that stochasticity in river flow has economy-wide implications, especially for economies like Egypt that are heavily dependent on rivers with large annual variations in flow. In such cases, it is necessary to consider hydrologic stochasticity in the ex-ante economic analysis of interventions in river systems, such as the operating strategy for new infrastructure. Coupling biophysical and economy-wide models is an improved approach for assessing direct, indirect, and induced macroeconomic impacts (e.g., GDP, employment, and income) of interventions in natural systems. Considering the coevolution of economic growth and resource supply and demand in simulating interventions in transboundary river systems enables analysts to better characterize the interdependencies between natural, engineered, and economic systems. This multi-sector dynamic analysis should be complemented with an assessment of the environmental and social effects that are not captured in macroeconomic indicators such as GDP^52,53^.

Water resources of the Nile River are limited and highly variable. Efficient utilization of Nile waters is key to the economic growth and resilience of its riparian states. This study demonstrates that a coordinated strategy for operating the GERD can reduce water deficits in Egypt, sustain Sudan’s Nile water use, and improve economic outcomes in Egypt and Ethiopia compared to an approach that resembles the Washington draft proposal. Although the results show increased economic outcomes to Egypt and Ethiopia from coordinated operation compared to the Washington draft proposal, we use different approaches and performance metrics to evaluate the economic impacts on the two countries.

The coordinated operation approach examined in this paper imposes only a small decline in total GERD electricity generation compared to an operation policy for the GERD that seeks to maximize firm power reliability (Supplementary Fig. 4). The benefits to Ethiopia from the coordinated operation of the GERD compared to the Washington draft proposal include an expected increase in total electricity generation. For Egypt, coordinated operation changes both irrigation water supply and hydropower generation. Overall, the CGE analysis indicates an expected positive impact on Egypt’s economy from coordinated operation as a result of reductions in irrigation deficits and changes in hydropower compared to the Washington draft proposal. Results show an increase in the present value of Egypt’s GDP is 76% of the simulated hydrological traces. Most of the increases in Egypt’s GDP occur in 5–15 years during multiyear periods of water scarcity when cooperation is needed the most (Supplementary Figs. 2 and 3). Agreement on a coordinated strategy could help facilitate regional water-energy-food-economy integration between the Eastern Nile Basin countries and achieve synergies that build on their comparative advantages (e.g., agriculture, hydropower, industry, and access to the sea). A multi-sector planning approach could increase resource use efficiency and the total regional economic gains.

Based on NBI data on future water infrastructures on the Eastern Nile, there is around 2.4 million hectares of undeveloped irrigation area in Ethiopia, Sudan, and South Sudan^54^. In this paper, we do not consider the impact of future irrigation expansion on water availability and hydropower generation in the Eastern Nile. However, future irrigation expansion in the Nile Basin in Ethiopia, Sudan, and South Sudan would reduce the Nile flow to Egypt.

Cooperation on transboundary rivers is not a binary decision but rather a continuum that can take place in different forms ranging between dispute and integration^55,56^. Cooperation often develops in small steps over long time periods to ensure mutual trust and political commitment^57^. Although the GERD is a hydroelectric project that does not consume water apart from reservoir evaporation and seepage losses (around 3.6 % of the annual flow of the Blue Nile on average), the negotiations on the initial filling and long-term operation of the dam are progressively becoming about Nile water allocation/sharing. The Nile water allocation issue has been outstanding for several decades, and attempting to address this issue within the GERD negotiations will make an agreement harder to achieve. While a deal on the initial filling and long-term operation of the GERD needs to be legally binding to provide assurance to Sudan and Egypt, it should be technically flexible and legally amendable to accommodate potential water allocation arrangements and future infrastructural development in Ethiopia. Seeking a technically flexible and legally amendable agreement on GERD initial filling and long-term operation could build trust between Ethiopia, Sudan, and Egypt and make negotiations on the more contentious water allocation issue more likely to succeed. The coordinated operation strategy described in this paper could be a positive step on this negotiation pathway and help foster a spirit of “neighbors looking out for each other.”

## Methods

### Structure of the modeling framework

The coevolutionary macroeconomy and river system simulation framework introduced in this study consists of two modeling components: (a) the Egyptian economy and (b) the Nile river system. The modeling framework accounts for the coevolutionary dynamics of river and economic systems using an iterative process. This multi-sector framework is designed for river systems with multiyear storage dams and a mix of hydro and non-hydro electricity generation. The two modeling components are described separately below, followed by a description of their interaction, which characterizes two-way hydro-economic feedbacks. The application of the coevolutionary framework to the Nile is then discussed.

### Economy-wide modeling component

The Egyptian economy-wide modeling component is based on the IFPRI (International Food Policy Research Institute) standard open-source CGE model^43^. The model was modified to include water, energy, and land components and run dynamically (i.e., for a multiyear period). In previous studies, water, energy, and land resources have been included in the productive activities of CGE models in a variety of ways. A recent review of the literature distinguished between CGE models that treat water as an explicit factor of production, those that include water as an implicit factor of production (i.e., embedded in land productivity), and those that treat water as a commodity (i.e., an intermediate input)^58^. Energy-oriented CGE models typically combine energy with capital in the production structure of goods and services^59,60^. The inclusion of energy in CGE models is straightforward compared to water because energy is a marketed commodity that can be easily reallocated to different sectors. The reallocation of water supplies across space and time requires storage and network infrastructure and is often constrained by unpredictable supplies (stochastic hydrology). Moreover, raw water supplies are typically unpriced^61–64^; thus, the economic value of water is not included in economic data (e.g., social accounting matrices and input–output tables).

In this study, we modified IFPRI’s standard CGE model such that economic activities produce commodities using a three-level process (Supplementary Fig. 5). At the top level, composite intermediate inputs and the value-added-energy bundle are combined to produce commodities using a Leontief Function^65^. The function maintains fixed proportions of inputs (composite intermediate inputs and value-added energy in this case) for each unit of output (commodity). At the second level, energy and value-added are aggregated using a Constant Elasticity of Substitution function (CES)^66^, such that the optimal input quantities of value-added and energy for each activity are determined based on relative prices subject to substitution elasticity similar to energy-oriented CGE models^59^. At the third level, substitution is allowed between the electricity commodity and other energy commodities using a CES function. A CES function is also used to combine labor, capital, and land into value-added.

The model is customized to allow each household group to allocate its consumption budget to the purchase of commodities based on a nested linear expenditure system (LES)^67^ and CES (Supplementary Fig. 5). At the top level, a LES function is used to divide the consumption budget between essential and nonessential demands^68^. The nonessential consumption budget is divided between five commodity categories using fixed shares. Each category includes different commodities that can substitute each other based on CES functions.

We modified the IFPRI CGE model to include four types of capital: (a) hydro capital used by a hydropower activity to produce electricity, (b) non-hydro capital used by a non-hydro activity to produce electricity, (c) water capital used by a municipal water activity to produce municipal water, and (d) general capital used by other activities. The use of land and water capital varies endogenously based on their rents. Logistic functions are used to simulate the response of the use of land and water capital to their rents. General and non-hydro capital grow based on past investments. Investment is allocated between these two capital types according to their relative rates of return. Given the increase in electricity demand resulting from economic growth, this specification of investment behavior allows for an incremental expansion of non-hydro electricity generation capacity; hydropower capacity does not grow endogenously with the year-to-year investment allocation. It is assumed that no new hydropower investments are made over the 30-year simulation period. To connect the economy-wide model with the river system model, dynamic exogenous shocks on land, water capital, hydro capital, and non-hydro capital are introduced to the economic model based on the river system modeling component, which simulates water and electricity availability, as explained below.

### River system modeling component

Pywr, a generic open-source Python library for simulating resource system networks^42^, is used to model the water system, including hydropower generation, in addition to an aggregated representation of non-hydro electricity generation. Pywr allows building resource system elements using input (e.g., catchments), output (e.g., water abstraction), and storage nodes (e.g., reservoirs). Nodes are linked in a network fashion to enable the flow and allocation of resources such as water and electricity. Pywr uses a time-stepping linear programming approach to drive resource allocation using priorities and system operating rules. Any time step resolution can be selected for Pywr simulations (e.g., hourly, daily, weekly, and monthly). Pywr’s multi-scenario simulation allows consideration of uncertainty in resource systems, e.g., hydrologic stochasticity.

The simulation results of Pywr can be processed, observed, and/or saved through “recorders.” We extended Pywr “recorders” to enable annual aggregation of the water and electricity metrics required for integration with the economy-wide modeling component. These metrics include annual irrigation and municipal water supply fractions, annual electricity generation from hydropower dams, and annual electricity generation from non-hydro energy generators.

### Coevolutionary macroeconomy and river system simulation

Supplementary Fig. 6 shows a flowchart of the novel coevolutionary macroeconomy and river system generalized hydro-economic^69^ modeling framework. The figure shows the interaction between the economy-wide modeling component (with an annual time step) and the river system modeling component (with a monthly time step) within each annual time step. Dynamic-recursive multiyear simulations are performed by repeating the procedure in Supplementary Fig. 6 multiple times.

The dynamic behavior of CGE models is typically driven by external drivers, such as capital growth (determined as a function of previous investment levels), labor growth, and productivity trends. In the first iteration, the CGE model solves based on its external drivers and determines changes to annual water and electricity demands and non-hydro electricity generation capacity relative to the economy’s initial year. Changes produced by the CGE model in relation to the irrigated area, the water capital, the demand for the electricity commodity, and the non-hydro capital are used as an estimate in the river system model for changes in irrigation water demand, municipal water demand, electricity demand, and non-hydro electricity generation capacity, respectively. The first CGE iteration assumes no irrigation deficit and electricity generation equal to that of the base year. The CGE and the river system models iteratively correct the initial assumptions of water curtailments and electricity generation, as explained in more detail below.

CGE models typically have an annual time step, but river system models run at smaller time intervals (e.g., monthly, weekly, daily, hourly). River system models have finer temporal resolutions to enable simulation of the spatial and temporal constraints of river basin resource systems, i.e., to better capture the consequences of stochastic hydrology and infrastructure constraints (e.g., reservoir storage). Although the iterative framework presented in Supplementary Fig. 6 is based on a monthly river system model, models with smaller time steps could also be used. The river system model uses the changes in irrigation water demand, municipal water demand, electricity demand, and non-hydro electricity generation capacity, computed by the economy-wide modeling component, to scale the corresponding water and electricity parameters. The river system model then performs a monthly simulation over a 12-month period based on monthly river flow data and the modified water and electricity demands and non-hydro capacity. The river system model then computes the fractions of annual irrigation and municipal water demands that can be met in addition to the annual hydro and non-hydro electricity generation. Water supply and electricity generation depend on the spatial and temporal availability of natural resources (e.g., river flow), infrastructure capacities (e.g., non-hydro and hydro capacities), and infrastructure operating rules.

After the river system modeling component determines water supply fractions and electricity generation, two tests are performed to determine (a) whether the models have converged and (b) when to stop iterating. These tests indicate whether to proceed to the next iteration or accept the current state of the CGE and the river system models as a solution for the annual time step. Passing either of the two tests terminates the iterative convergence process. The CGE and the river system models pass the convergence test when the difference between the current and the previous iteration’s values of an annual economy, water, or energy metric falls below a certain convergence threshold. The value of the threshold depends on the desired level of accuracy and available computational capacity. The stopping test imposes a maximum number of iterations at which the current state of the CGE and the river system models is considered a solution for the annual time step. The stopping test acts as a safeguard to prevent excessively long iteration over one annual time step. The convergence test is performed starting from the second iteration. Thus, at least two iterations are performed within each annual time step to ensure convergence.

Failure in the convergence and stopping tests results in proceeding to the next iteration. In the next iteration, annual water supply fractions and electricity generation of the previous iteration are applied to the CGE model to compute new changes to annual water and electricity demands and non-hydro electricity generation capacity relative to the initial year of the economy (i.e., the base year). The irrigation and municipal water supply fractions, computed by the river system modeling component, are introduced to the CGE model as shocks to the land and water capital, respectively. The ratio between current electricity generation and electricity generation in the initial year of the economy is calculated for each of the two electricity generation technology groups (i.e., hydro and non-hydro) and introduced as shocks to the hydro and non-hydro capitals.

### Implementation of the coevolutionary framework

The open-source Python Network Simulation (Pynsim) framework^44^ was extended and used to integrate the economy-wide and river system modeling components and to manage their iteration, sequencing, and time stepping. Each of the two components was specified as a Pynsim “engine”^44^. Although the IFPRI CGE model is written in the General Algebraic Modeling System (GAMS)^70^, it was linked to Pynsim through the GAMS Python Application Programming Interface. Eight Pynsim integration nodes were created for data exchange between the economy-wide and river system modeling components. Four of the integration nodes transfer changes in annual water (irrigation and municipal) and electricity demands and non-hydro electricity generation capacity from the economy-wide to the river system modeling components. The other four integration nodes transfer the annual water (irrigation and municipal) supply fractions and hydro and non-hydro electricity generation from the river system to the economy-wide modeling components.

### Eastern Nile River system model

Supplementary Fig. 7 shows a schematic of the monthly river system model of the Eastern Nile Basin. The model uses naturalized inflow data for the period 1901–2002, obtained from the Eastern Nile Technical Regional Office^54^. The Eastern Nile River System model contains all major dams and water consumers in the basin, including the GERD and the HAD. The baseline water withdrawal targets are shown in Supplementary Fig. 8. Supplementary Table 1 reports the main characteristics of the dams included in the Nile River System model. The model was calibrated and validated at eight locations across the basin based on historically observed river flows and reservoir water levels over 1970–2002. This period was chosen based on the availability of observed data. Supplementary Fig. 9 and Supplementary Table 2 show the performance of the Eastern Nile River system at eight locations. In the model, non-hydro electricity generation is used to fill the gap between hydropower generation and electricity demand, subject to generation capacity. This assumption is valid since hydropower in Egypt is a by-product of other activities. Furthermore, the historical evolution of the Egyptian electricity mix shows relatively regular annual hydropower generation with a steady increase in electricity generation from other technologies to fill the supply-demand gap^8^.

### Initial filling assumptions of the Washington draft proposal

Supplementary Table 3 describes the 5-year plan for the initial filling of the GERD in the Washington draft proposal assuming normal or above-average hydrological conditions. We assumed that after achieving the water retention target of the first year (4.9 bcm), two 375 MW turbines become operational. The rest of the turbines become operational after achieving the second year’s water retention target (18.4 bcm). We assumed that once the filling targets of year-1 or year-2 are achieved, reservoir storage is always maintained above these targets in order to keep the turbines operational. In the Washington draft proposal, water retention is limited to July and August, with a minimum environmental release of 43 Mm^3^/day. During the initial filling period, from September to June, releases from the GERD equal the inflow to the reservoir. However, if a drought occurs during the 5-year initial filling plan specified in Table S3, the Washington draft proposal has provisions for implementing delays in filling the GERD (our assumptions for these provisions are described in a later section).

### Long-term operation assumptions of the Washington draft proposal

The Washington draft proposal’s operating rules for the long-term operation of the GERD begins when reservoir storage reaches 49.3 bcm. We assumed that when reservoir storage is at or above 49.3 bcm, water is released through the GERD’s turbines to maintain a constant monthly energy production of 1170 GWh to maximize the 90% power generation reliability^71^. If reservoir storage drops below 49.3 bcm, the target monthly energy production is reduced to 585 GWh. The purpose of reducing the energy generation target is to enable the GERD storage to recover above 49.3 bcm. Water releases designed to maintain a regular power rate depend on the reservoir water level at the beginning of the time step (the higher the water level, the lower the releases required). A minimum environmental release of 43 Mm^3^/day is maintained throughout the year when possible. Additional water releases may be made following drought mitigation mechanisms that resemble those of the Washington draft proposal, as described below.

### Drought mitigation assumptions of the Washington draft proposal

The Washington plan includes three mechanisms to mitigate the adverse effects of droughts, prolonged droughts, and prolonged periods of dry years on the downstream riparians^46^. The mechanism for mitigating droughts is triggered when the GERD’s annual inflow is forecast to be ≤37 bcm. This first mechanism requires Ethiopia to release a minimum annual water volume, depending on the forecast annual inflow and GERD storage at the beginning of the hydrologic year (see Exhibit A in Egypt’s letter to the United Nations Security Council dated 19 June 2020^46^).

The effectiveness of the mechanism for mitigating droughts depends on the accuracy of the forecast of the annual inflow for the upcoming hydrological year. To implement the Washington plan in this study’s river simulation model, we do not forecast annual flows for the next hydrological year. Instead, drought mitigation conditions are checked in March of every hydrologic year, by which time, on average, about 96% of the river’s annual flow is already known because it occurs from June to February. If necessary, water releases during the remaining 3 months of the hydrological year (March–May) are increased to achieve the minimum annual releases specified in the mechanism for mitigating droughts. These increased releases during March–May effectively offset any deviations from water releases specified by the drought mitigation mechanism given the dam inflows and releases in the previous 9 months of the current hydrologic year.

The mechanism for mitigating prolonged droughts requires that the average annual release over every 4-year period equal at least 39 bcm (37 bcm during the initial filling). In the implementation of this prolonged drought mitigation mechanism of the Washington draft proposal in our river simulation model, we check in March of every hydrological year to ensure that this annual average release over the previous 4-year period is achieved. Although this mechanism does not depend on reservoir inflow, it is also checked for in March to provide flexibility to GERD operation during the rest of the year.

The mechanism for mitigating prolonged periods of dry years is similar to the prolonged drought mitigation mechanism, except the period over which annual releases are averaged is longer (5 years) and the average annual release is higher (40 bcm). We implement this mechanism in our river simulation model in the same way, checking in March of every hydrological year to ensure that the annual average release over the previous 5-year period is achieved. Supplementary Fig. 10 shows the exceedance probability of the annual, 4-year average annual, and 5-year average annual flow of Blue Nile at the location of the GERD over the period 1901–2002. The drought mitigation thresholds of the Washington draft proposal are marked in the figure to show their probability of occurrence in the river flow data.

If a deficit from the minimum releases of any of the three mechanisms is identified at the beginning of March, water releases over March–May are increased equally in each month to offset the deficit.

### Initial filling assumptions of the coordinated operation

The coordinated operating strategy for the initial filling of the GERD is similar to the Washington plan, except for the retention of inflows to meet the targets in Table S3 is not constrained to July and August. The coordinated operation requires that a minimum environmental release of 43 Mm^3^/day be maintained throughout the year when possible. If physically possible, releases from the GERD are also greater than or equal to (1) Sudan’s monthly water withdrawal targets along the Blue and Main Nile, plus (2) Egypt’s monthly water release target from the HAD if HAD storage is below 50 bcm (156 m a.s.l.). This operating strategy enables Ethiopia to avoid delays in filling the GERD as long as HAD storage is at or above 50 bcm. In simulating coordinated operation, the operations of the Roseires, Sennar, and Merowe dams have been adapted to pass GERD releases intended to benefit Egypt. It was assumed that two of the GERD turbines become operational after achieving the first year’s water retention target, and the rest of the turbines become operational once the second year’s filling target is achieved. After achieving the filling targets of year-1 or year-2, reservoir storage is always maintained above these targets (i.e., 4.9 or 18.4 bcm) to keep the turbines operational.

### Long-term operation assumptions of the coordinated operation

As with the Washington draft proposal, the long-term operation of the GERD begins as soon as reservoir storage reaches 49.3 bcm. Also the same as the Washington plan, it was assumed that when reservoir storage is at or above 49.3 bcm, water is released through the GERD’s turbines to maintain a constant monthly energy production of 1170 GWh to maximize the 90% power generation reliability^71^. If reservoir storage drops below 49.3 bcm, the target monthly energy generation is reduced to 585 GWh. A minimum environmental release of 43 Mm^3^/day is maintained throughout the year when physically possible. The key difference between the Washington draft proposal and coordinated operation is that when physically possible, the coordinated operation ensures that the GERD releases are greater than or equal to Sudan’s water withdrawal targets on the Blue and Main Nile plus Egypt’s target releases from the HAD if HAD storage is below 50 bcm (156 m asl). This provides Ethiopia more flexibility in the operation of the GERD as long as HAD storage is at or above 50 bcm.

### Drought mitigation assumptions of the coordinated operation

The coordinated operation strategy does not include drought mitigation measures that are based on minimum annual water releases. Instead, a dynamic mechanism is used to help reduce downstream water deficits during periods of water scarcity, as explained in previous sections. Such an approach provides flexibility to Ethiopia in GERD operation and increases the basin-wide and national water, electricity, and economic gains.

### Economy-wide model of Egypt

The CGE model of Egypt represents a dynamic-recursive, single-country, open-economy, including four agent types: households, enterprises, the government, and the rest of the world. Households are classified into ten groups based on location (urban or rural) and income (five quintiles). The model includes 11 production activities: agriculture, light industry, heavy industry, construction, transport, hydropower, non-hydro, other energy, municipal water supply, public services, and other services. Each of the 11 activities produces a distinct commodity except hydropower and non-hydro, which produce a similar commodity (i.e., electricity). Production activities use six factors of production to produce commodities: labor, land, general capital, water capital, hydro capital, and non-hydro capital. Labor and general capital are assumed to be mobile across production activities, whereas land, water capital, hydro capital, and non-hydro capital are specific to agriculture, municipal water supply, hydropower, and non-hydro, respectively. Labor is updated exogenously to follow the projected changes in the 16–64 age group of the shared socioeconomic pathways (SSPs) “middle of the road” scenario^72^. Total factor productivity is also updated exogenously to follow economic performance under the “middle of the road” scenario.

The CGE model of Egypt assumes fixed price of commodities on the international market following the small open-economy assumption, i.e., that the economy participates in international trade but does not affect world prices^73^. Government spending is simulated as a fixed share of total absorption (total demand for marketed goods and services). The model follows the saving-investment identity (savings are equal to investment) assuming fixed saving propensities. Foreign savings are assumed fixed, and the exchange rate is flexible.

The baseline model was calibrated to a 2019 Social Accounting Matrix (SAM) of Egypt. The 2019 SAM was generated based on a 2011 SAM using an expansion factor equal to the ratio between the Egyptian GDP in the 2 years. We compared the generated SAM with the structure of Egypt’s economy based on the most recent data in the World Bank Database; no significant differences were found in the economy’s structure. Supplementary Fig. 11 shows this comparison.

### Nile River system–Egypt’s economic integration

The Eastern Nile River system model and the CGE model of Egypt run dynamically over a 30-year simulation period (2020–2049) and multiple scenarios. For each 30-year simulation, the CGE model executes 30 annual time steps, and the river system model executes 360 monthly time steps (30 years × 12 months). The CGE and river system models are integrated through the water and electricity sectors, as described earlier. The convergence test is performed using the GDP at market prices with an assumed convergence threshold of US$ 5 million. A maximum of 50 iterations is specified for each annual time step. All simulated time steps converged in <50 iterations.

## Supplementary information

Supplementary Information

## Data Availability

The data that support the findings of this study are available from the corresponding author upon reasonable request at Zenodo: 10.5281/zenodo.4314574. The corresponding author will make the economy-related data available upon reasonable request. The  Eastern Nile River system model and its data are not publicly available due to state restrictions and contain information that could compromise research participant privacy/consent.

## Code availability

The Pywr and Pynsim software libraries used to develop the integrated economy-wide and river system model are open-source and freely available in the following repositories: https://github.com/pywr/pywr; https://github.com/UMWRG/pynsim. The standard CGE model of IFPRI is freely accessible through the following link: https://www.ifpri.org/publication/standard-computable-general-equilibrium-cge-model-gams-0.
