# Supplementary material for: Collaborative management of the Grand Ethiopian Renaissance Dam increases economic benefits and resilience
Source: Nat Commun. 2021 Sep 23;12:5622. doi: 10.1038/s41467-021-25877-w (PMC8460785; doi:10.1038/s41467-021-25877-w)
Supplement: Supplementary file 1 — Supplementary Information [file 41467_2021_25877_MOESM1_ESM.pdf]

Supplementary Information for

Collaborative management of the Grand Ethiopian Renaissance Dam increases economic benefits and resilience

Mohammed Basheer [1], Victor Nechifor [2], Alvaro Calzadilla [2], Khalid Siddig [3, 4], Mikiyas Etichia [1], Dale Whittington [5, 6], David Hulme [5], Julien J. Harou [1, 7, *]

[1] Department of Mechanical, Aerospace and Civil Engineering, The University of Manchester, Manchester, UK.

[2] Institute for Sustainable Resources, University College London, London, UK.

[3] International Agricultural Trade and Development, Humboldt-Universität zu Berlin, Berlin, Germany.

[4] Department of Agricultural Economics, University of Khartoum, Khartoum, Sudan.

[5] Global Development Institute, The University of Manchester, Manchester, UK.

[6] Departments of Environmental Sciences & Engineering and City & Regional Planning, University of North Carolina, Chapel Hill, NC, USA.

[7] Department of Civil, Environmental and Geomatic Engineering, University College London, London, UK.

*Correspondence to: julien.harou@manchester.ac.uk

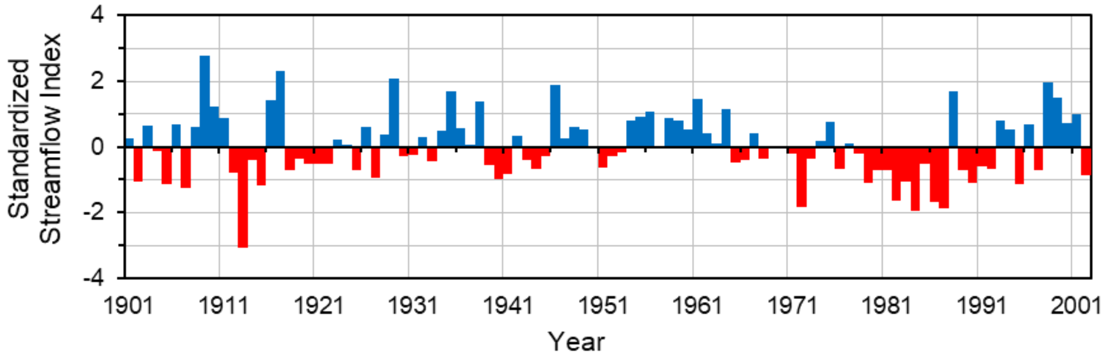

Supplementary Figure 1. Standardized Streamflow Index of the annual flow of the Blue Nile near the Ethiopian-Sudanese border. The blue and red colors indicate above- and below-normal annual flow values, respectively.

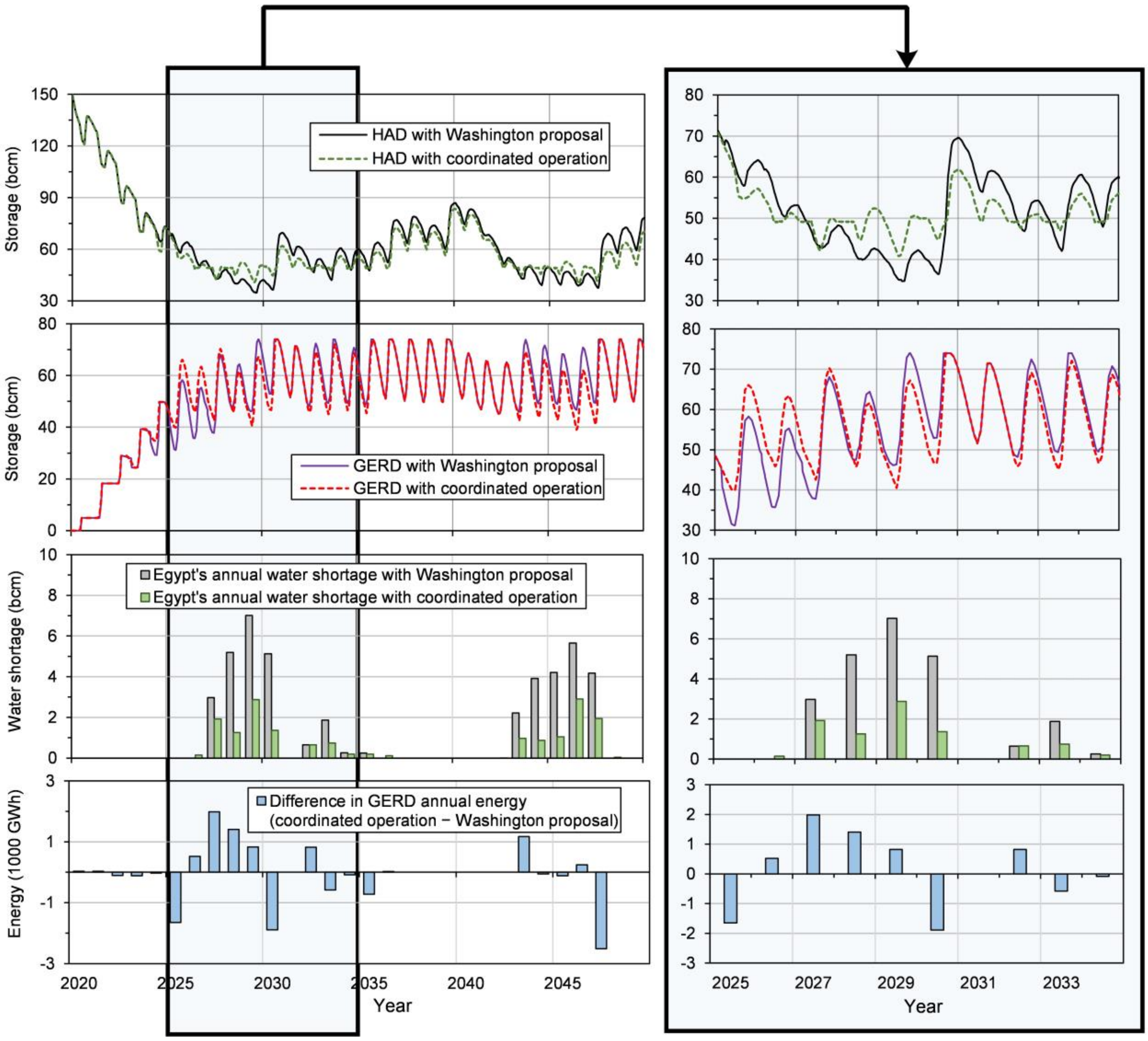

Supplementary Figure 2. A River flow sequence with the highest Egyptian benefit from coordinated operation compared to the Washington proposal.

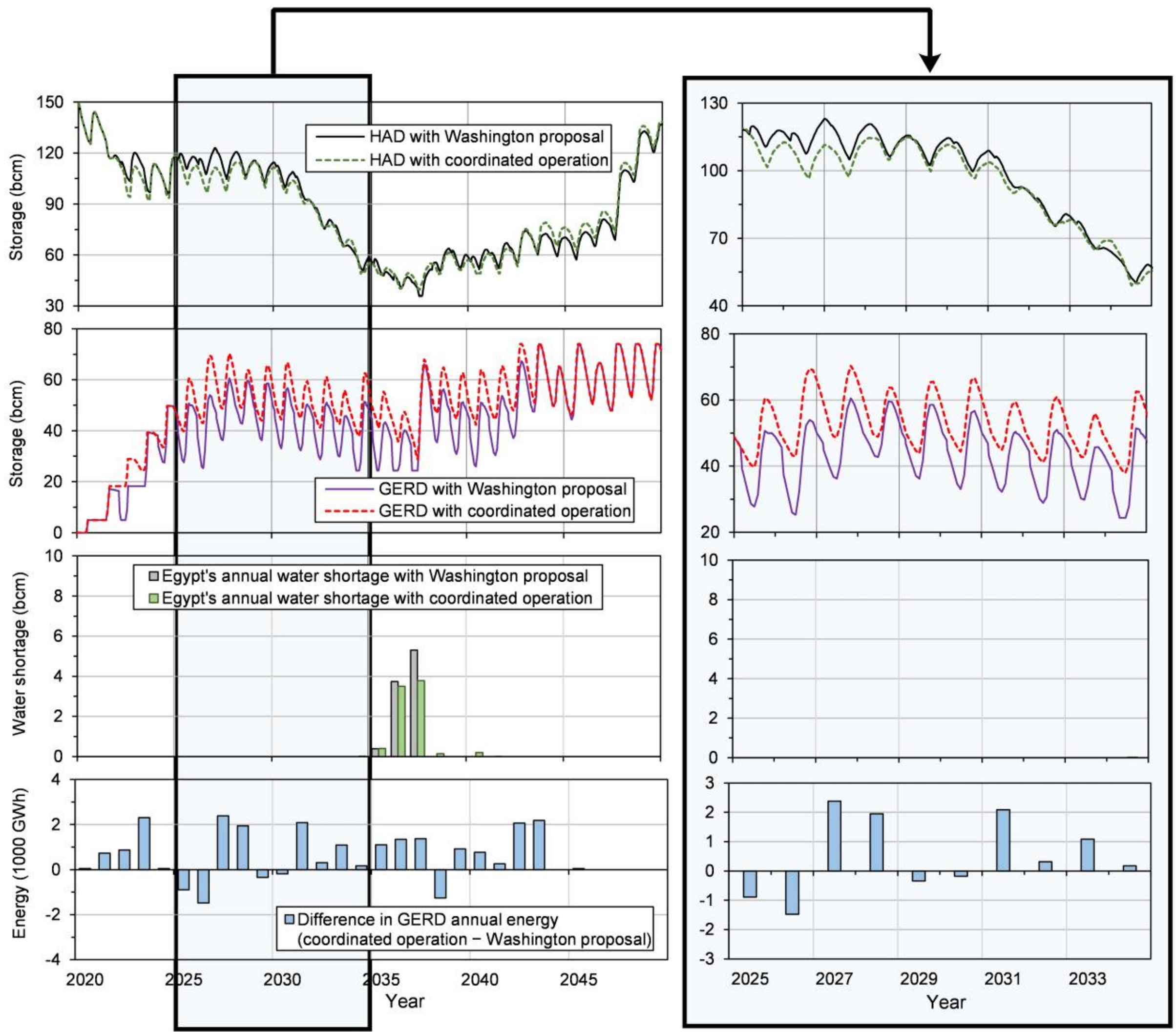

Supplementary Figure 3. A River flow sequence with the highest Ethiopian benefit from coordinated operation compared to the Washington proposal.

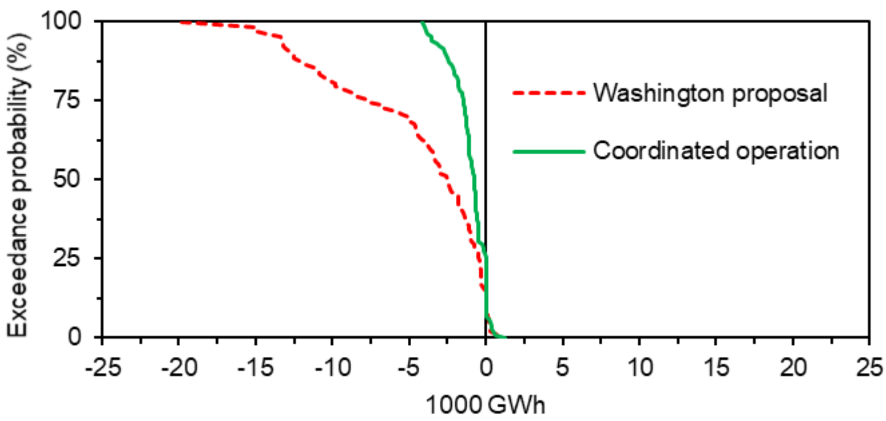

Supplementary Figure 4. Deviation of GERD accumulated energy generation under the Washington Proposal and coordinated operation from the dam's accumulated energy with an operating rule aims at maximizing the 90% power reliability.

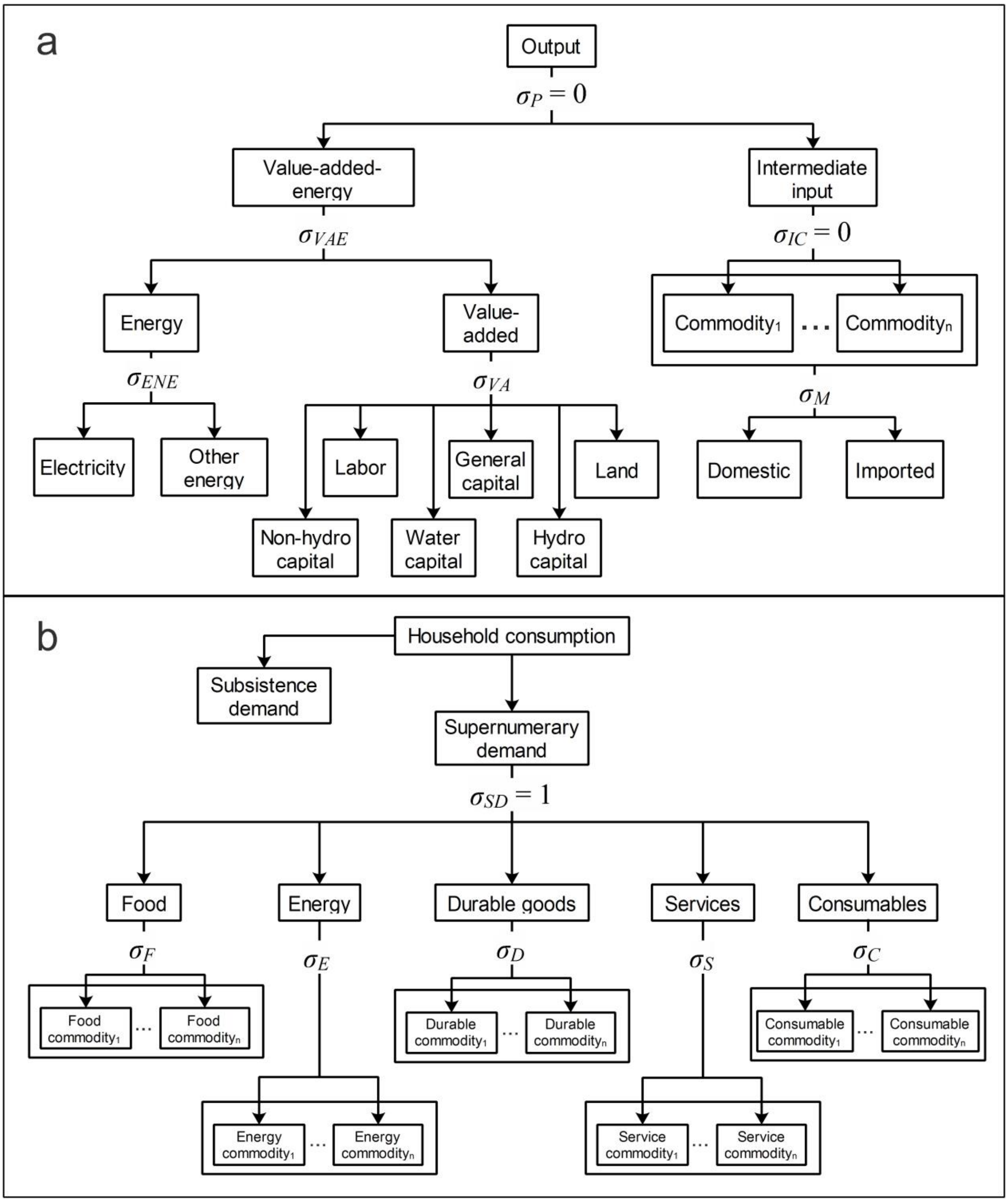

Supplementary Figure 5. Production and consumption in the Computable General Equilibrium. a Specification of production. **b** Specification of household consumption. σ stands for elasticity of substitution.

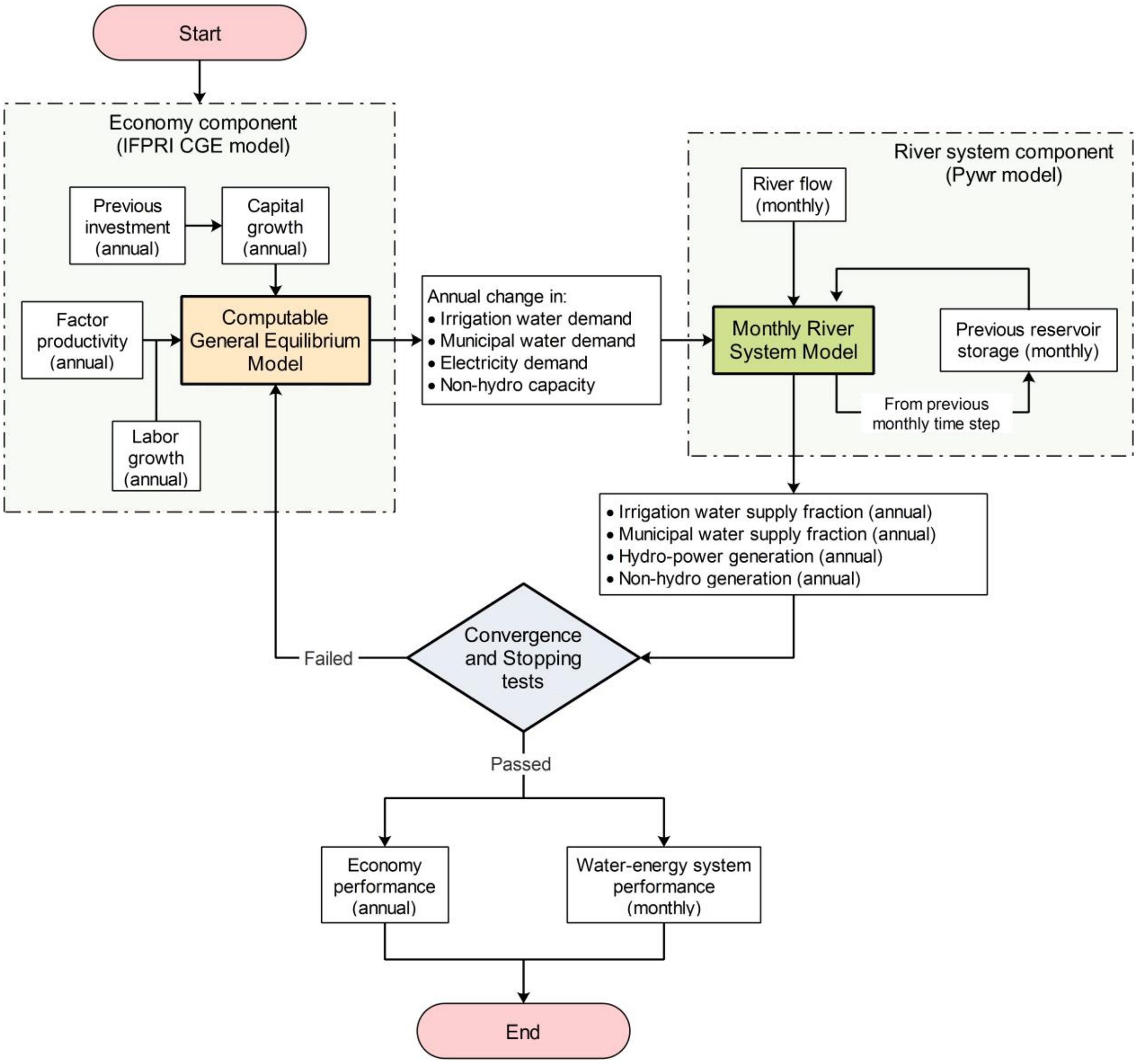

Supplementary Figure 6. Co-evolutionary macro-economy and river system modeling framework. The framework integrates a monthly river system model with an annual Computable General Equilibrium (CGE) model. The figure represents the interactions between the two models within one annual time step.

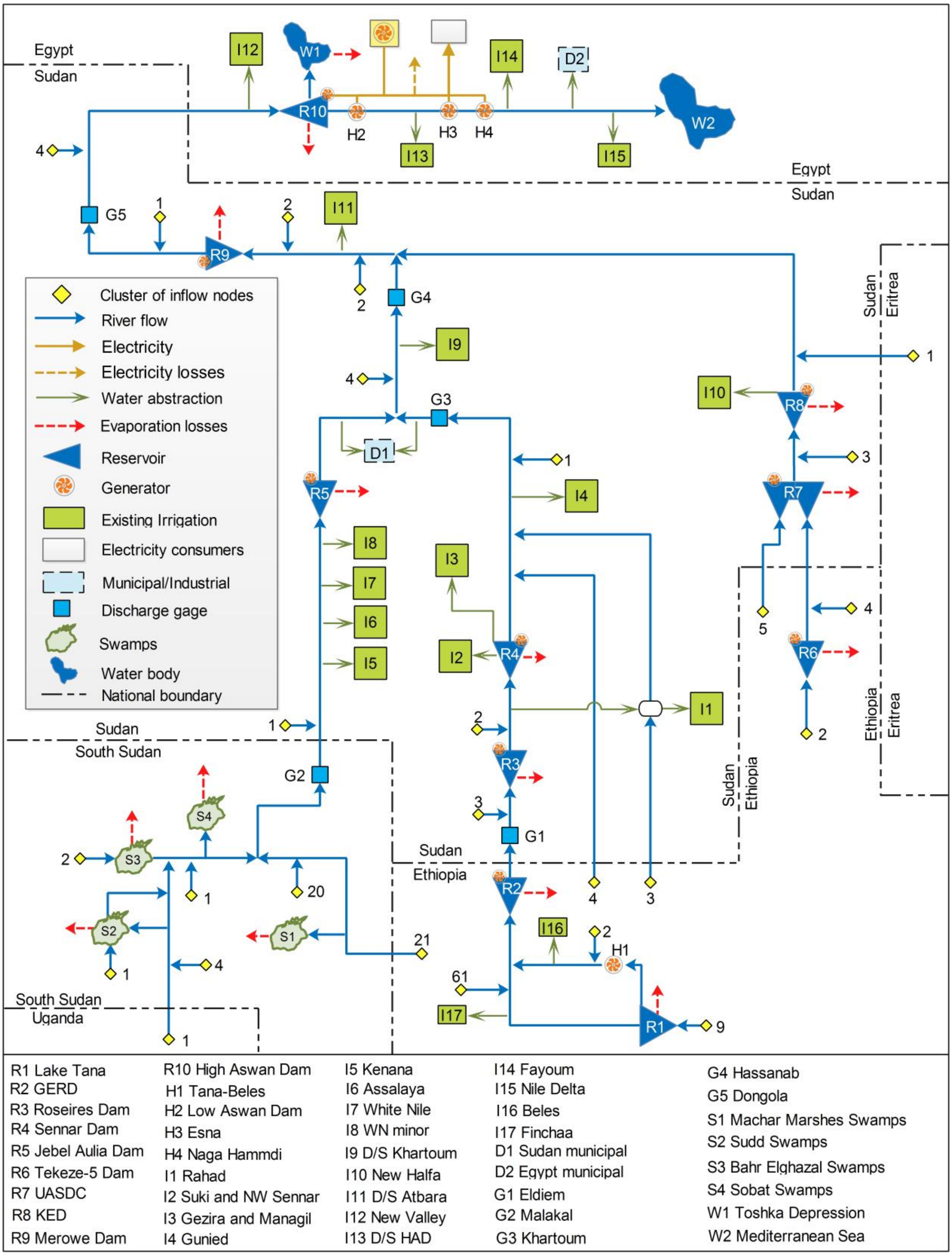

Supplementary Figure 7. Schematic of the monthly Eastern Nile River Simulation Model. For visualization purposes, the inflow nodes were clustered into groups, with the number next to each cluster representing the number of clustered nodes. GERD stands for Grand Ethiopian Renaissance Dam, UASDC stands for Upper Atbara and Setit Dam Complex, KED stands for Khashm Elgirba Dam, D/S stands for downstream, and HAD stands for High Aswan Dam.

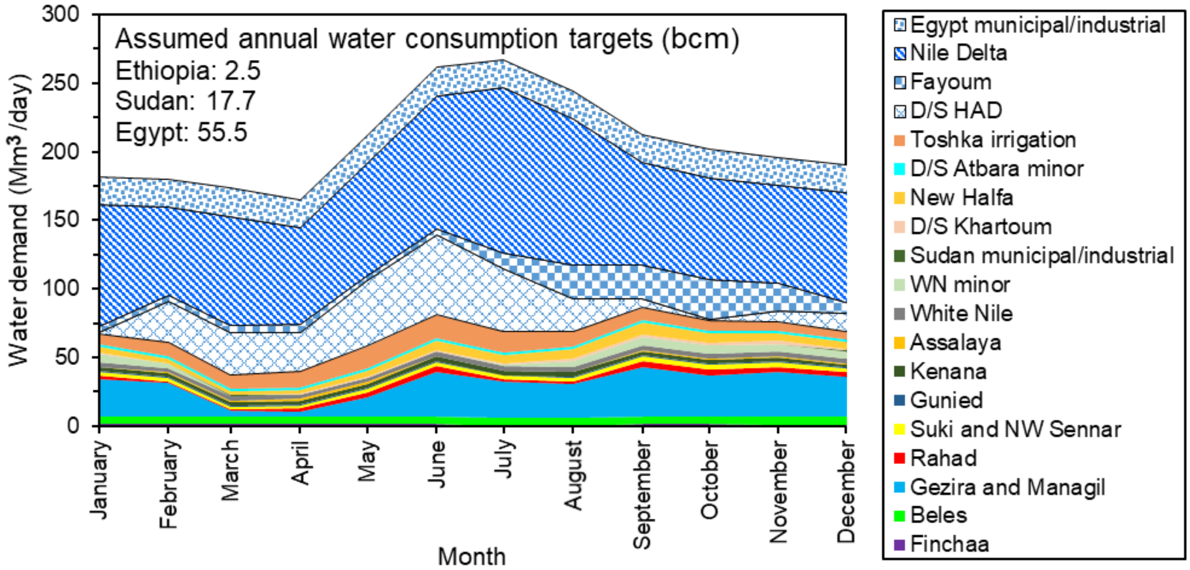

Supplementary Figure 8. Assumed water consumption targets of the current major consumers of the Eastern Nile river flow. Water deficits are measured from these targets. D/S stands for downstream, and HAD stands for High Aswan Dam.

Supplementary Table 1. Main features of the dams in the Eastern Nile river system model.

Dam	FSL (masl)	MOL (masl)	Storage at FSL (bcm)	Storage type	Hydropower capacity (MW)
GERD	640	590	74	Multi-year	5,150
Roseires	490	469	5.9	Seasonal	280
Sennar	421.7	417.2	0.64	Seasonal	15
Jebel Aulia	377.4	372.5	3.18	Seasonal	30.4
Tekeze-5	1,140	1,096	9.3	Multi-year	300
UASDC	521	509	3.69	Seasonal	320
KED	474	473	0.63	Seasonal	16
Merowe	300	285	12.4	Seasonal	1,250
HAD	185	147	182.7	Multi-year	2,100
Finchaa	2,220	2,214	1.3	Seasonal	100
Tana Beles	-	-	-	Run-of-the-river	423
LAD	-	-	-	Run-of-the-river	315
Esna	-	-	-	Run-of-the-river	90
Naga Hammdi	-	-	-	Run-of-the-river	64

Note: FSL = Full Supply Level; MOL = Minimum Operating Level; GERD = Grand Ethiopian Renaissance Dam; UASDC = Upper Atbara and Setit Dam Complex; KED = Khashm Elgirba Dam; HAD = High Aswan Dam; LAD = Low Aswan Dam

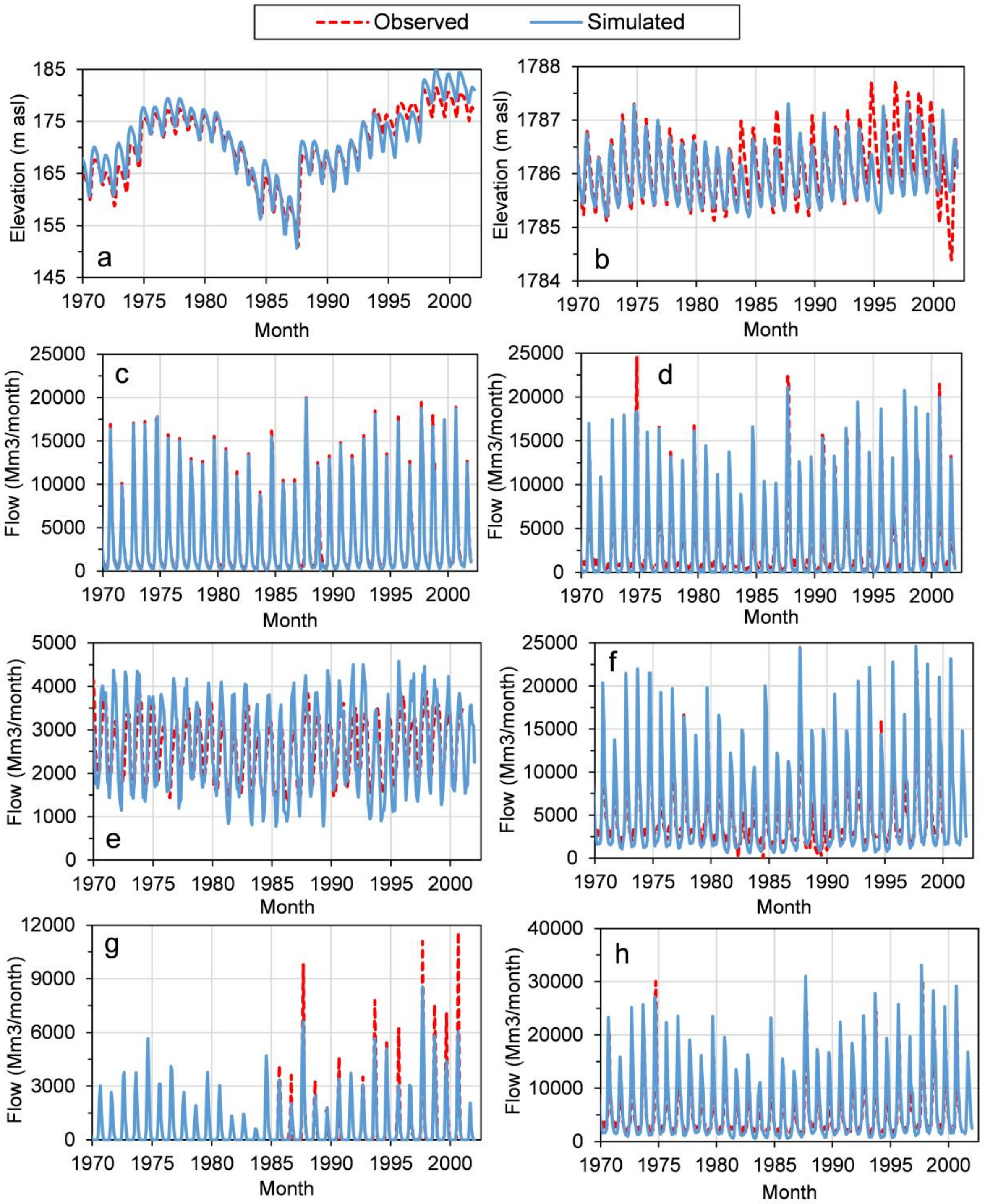

Supplementary Figure 9. Performance of the Eastern Nile River system model at different locations. a High Aswan Dam reservoir water level. **b** Lake Tana water level. **c** Eldiem Gage flow. **d** Khartoum Gage flow. **e** Malakal Gage flow. **f** Hassanab Gage flow. **g** Khashm Elgirba Dam outflow. **h** Dongola Gage flow.

Supplementary Table 2. Eastern Nile River System model performance in the calibration and validation periods.

Parameter	Location	Performance metric	Calibration (1970-1986)	Validation (1987-2002)
Flow	Eldiem Gage	NSE	0.99	0.99
		R^2	0.99	0.99
	Malakal Gage	NSE	0.17	0.24
		R^2	0.61	0.65
	Khartoum Gage	NSE	0.89	0.91
		R^2	0.93	0.92
	Hassanab Gage	NSE	0.69	0.71
		R^2	0.87	0.86
	Khashm Elgirba Dam	NSE	0.72	0.76
		R^2	0.81	0.75
	Dongola Gage	NSE	0.81	0.83
		R^2	0.88	0.89
Water level	Lake Tana	NSE	0.79	0.45
		R^2	0.80	0.52
	High Aswan Dam	NSE	0.86	0.87
		R^2	0.91	0.90

Note: NSE = Nash-Sutcliffe efficiency; R^2 = coefficient of determination

Supplementary Table 3. GERD stage-based filling plan.

Stage	Hydrologic year	Water volume retained (bcm)	Cumulative water volume retained (bcm)
1	1	4.9	4.9
	2	13.5	18.4
2	3	10.5	28.9
3	4	10.4	39.3
4	5	10.0	49.3

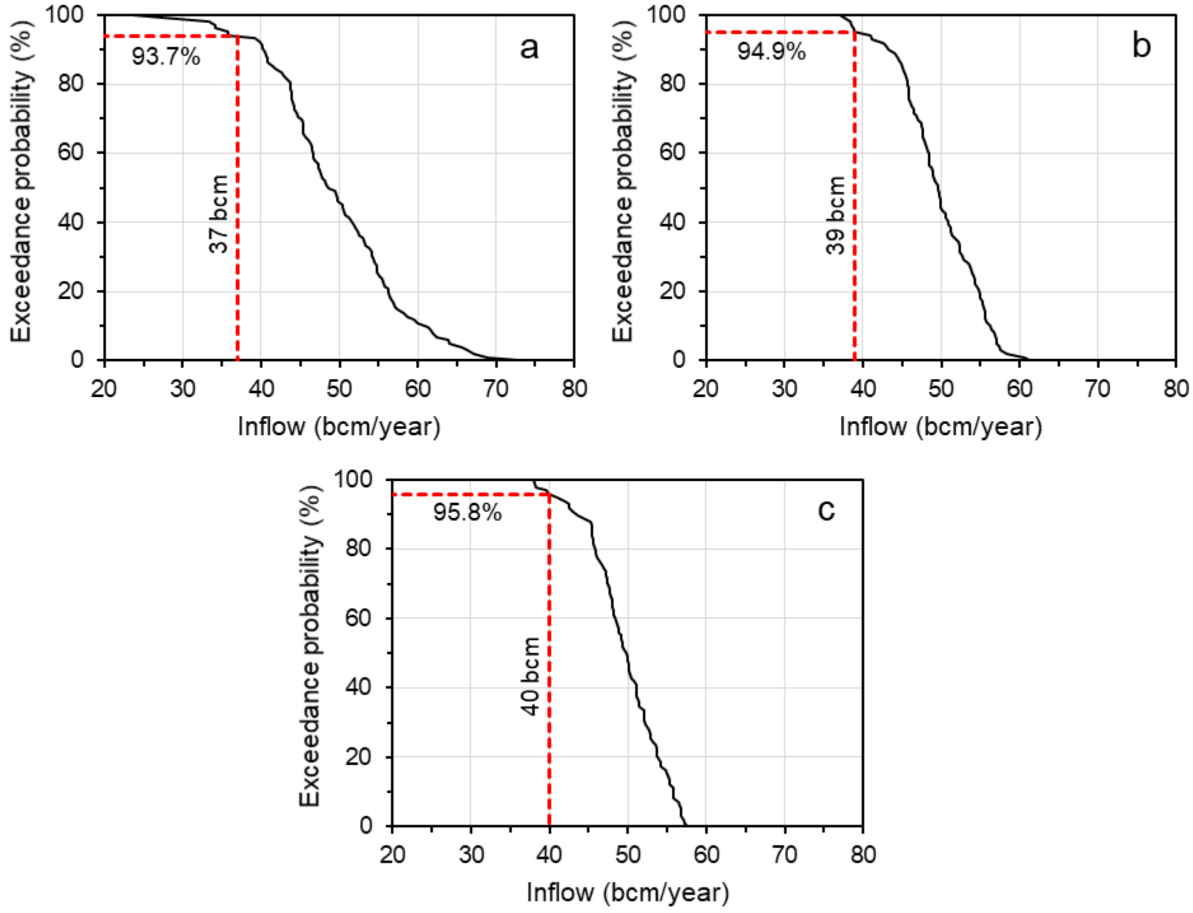

Supplementary Figure 10. Exceedance probability of the Blue Nile flow at the location of the Grand Ethiopian Renaissance Dam (GERD) over the period 1901-2002. a Annual flow. **b** Four-year mean annual flow. **c** Five-year mean annual flow. The dashed lines mark the GERD outflow thresholds of the Washington draft proposal.

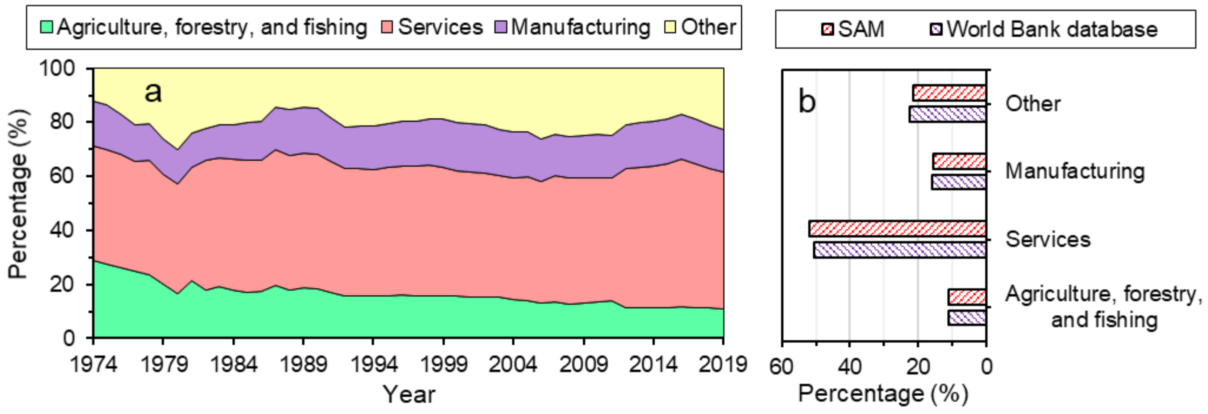

Supplementary Figure 11. Egypt's percentage sectoral contributions to Gross Domestic Product at factor cost. a Time series based on the World Bank database. **b** Comparison between the World Bank database and the Social Accounting Matrix (SAM) used in this study for the year 2019.
